# Nursing Challenges in Iran

**Published:** 2014-06-15

**Authors:** Ismail Azizi Fini

**Affiliations:** 1 Department of Critical Care Nursing, School of Nursing and Midwifery, Tehran University of Medical Sciences, Tehran, IR Iran

**Keywords:** Challenge, Nursing, Iran

Dear Editor,

In a recent issue of the journal, you published an article about enhancing nursing students’ transition to workplace. The author mentioned that during transition, nurses frequently encounter role conflict, marginalization and burnout. These problems may lead to job dissatisfaction, intention to leave their job and also may interfere with quality of care they provide (1). In another article in the same issue, Ghorbani et al. (2) evaluated public and private hospital nurses’ perceptions of the ethical climate in their work settings. The latter paper reported that managerial factors were the most important factors affecting the ethical climate both in public and private hospitals. Both the nurses’ transition to work place and the managerial factors affecting nursing care are among the challenging issues in nursing in Iran and other countries. Several other papers in this journal and other journals have also addressed nurses’ challenges in application of their knowledge and skills in practice (3, 4), educational and societal barriers faced with men in nursing such as their marginalization and not acceptance within nursing as a female career (5). It seems that there are basic challenges in nursing profession in Iran and some other countries. Method of entrance in nursing profession (6), nurses’ lack of political power and lack of participation in important organizational and national health policies (7, 8), insufficient supports from associations, financial problems, non-professional activities of associations and lack of interactions among nursing associations are among the main problems nurses face in Iran (9). Among these problems, I think the inappropriate method of entrance in nursing is the cornerstone of challenges, which made nurses ineffective and dissatisfied in Iran and prevented them to be a real “force for change”.

In Iran, university entrance tests, a non-specific theoretical and aptitude test, is the only criterion for entrance of applicants into all academic professions. Applicants with higher scores enter medicine and dentistry fields, while those with relatively lower scores enter nursing and other fields. No other features are assessed while studies showed that caring professions need appropriate psychological and personal characteristics in addition to their knowledge and aptitude. Applicants usually enter a special field of study and then a profession without any information about it (4). Therefore, many nursing students do not have a high-level motivation for being in the profession and entering clinical practice. Such problem is also increasing when they face with nurses poor work conditions, high workload, low salary and limited clinical autonomy. Then, they do not perform with required standards in practice, experience psychological pressures and finally leave their job. Some although remain, but become indifferent and motiveless and make the working environment tedious (10). This is why I as a nurse educator in line with several nurse researchers believe that some steps must be performed for improving nurses financial and working conditions, simultaneously the method of entrance in to the nursing profession and student selection should be revised. In addition there is no mechanism for evaluating competencies of nursing graduates in Iran. While some studies showed that nursing graduates lack clinical competencies (11). Then, interventions to overcome these challenges seem necessary.
